# Acute effects of ambient temperature and particulate air pollution on fractional exhaled nitric oxide: A panel study among diabetic patients in Shanghai, China

**DOI:** 10.1016/j.je.2017.01.002

**Published:** 2017-06-20

**Authors:** Huichu Li, Hongjian Bai, Changyuan Yang, Renjie Chen, Cuicui Wang, Zhuohui Zhao, Haidong Kan

**Affiliations:** a School of Public Health, Key Lab of Public Health Safety of the Ministry of Education and Key Lab of Health Technology Assessment of the Ministry of Health, Fudan University, Shanghai, China; b The First People's Hospital of Yancheng, Jiangsu Province, China; c Shanghai Key Laboratory of Atmospheric Particle Pollution and Prevention (LAP^3^), Fudan University, Shanghai, China

**Keywords:** Temperature, Particulate matter, Fractional exhaled nitric oxide, Respiratory inflammation, Panel study

## Abstract

**Background:**

Epidemiological studies have shown the associations of ambient temperature and particulate matter (PM) air pollution with respiratory morbidity and mortality. However, the underlying mechanisms have not been well characterized. The aim of this study is to investigate the associations of temperature and fine and coarse PM with fractional exhaled nitric oxide (FeNO), a well-established biomarker of respiratory inflammation.

**Methods:**

We conducted a longitudinal panel study involving six repeated FeNO tests among 33 type 2 diabetes mellitus patients from April to June 2013 in Shanghai, China. Hourly temperature and PM concentrations were obtained from a nearby fixed-site monitoring station. We then explored the associations between temperature, PM, and FeNO using linear mixed-effect models incorporated with distributed lag nonlinear models for the lagged and nonlinear associations. The interactions between temperature and PM were evaluated using stratification analyses.

**Results:**

We found that both low and high temperature, as well as increased fine and coarse PM, were significantly associated with FeNO. The cumulative relative risk of FeNO was 1.75% (95% confidence interval [CI], 1.04–2.94) comparing 15 °C to the referent temperature (24 °C) over lags 0–9 days. A 10 μg/m^3^ increase in fine and coarse PM concentrations were associated with 1.18% (95% CI, 0.18–2.20) and 1.85% (95% CI, 0.62–3.09) FeNO in lag 0–1 days, respectively. PM had stronger effects on cool days than on warm days.

**Conclusions:**

This study suggested low ambient temperature, fine PM, and coarse PM might elevate the levels of respiratory inflammation. Our findings may help understand the epidemiological evidence linking temperature, particulate air pollution, and respiratory health.

## Highlights

• Both low and high temperatures were significantly associated with FeNO.
• The increases of fine and coarse PM concentrations were associated with FeNO.
• Both fine and coarse PM had stronger effects in cool days.

## Introduction

A number of epidemiological studies have linked short-term variations in temperature and particulate matter (PM) air pollution with respiratory morbidity and mortality.^1^, ^2^ However, the underlying mechanisms responsible for these associations have not been well characterized. Inflammation constitutes a key pathway in the development and exacerbations of respiratory diseases. Fractional exhaled nitric oxide (FeNO) is recommended by the American Thoracic Society as a non-invasive biomarker of airway inflammation and has been widely used in clinical practice and epidemiological studies of respiratory diseases.^3^, ^4^, ^5^

FeNO has been associated with PM exposure in China and other parts of the world.^6^, ^7^, ^8^, ^9^, ^10^ Previous investigators have proposed that PM's effects increase with smaller sizes.^11^ However, recent toxicological studies suggested a greater inflammatory potential in the coarse fraction of PM (PM_10-2.5_) than in the fine fraction of PM (PM_2.5_),^12^, ^13^ and few population-based studies has evaluated the effects of PM_10-2.5_ on FeNO. On the other hand, to the best of our knowledge, there were no studies investigating the association between ambient temperature and FeNO, leading to a lack of biological support to the observed association between temperature variations and respiratory mortality and morbidity.

Therefore, we conducted a longitudinal panel study to investigate the associations of ambient temperature, PM_2.5_, and PM_10-2.5_ with FeNO in a group of type 2 diabetes mellitus (T2DM) patients in Shanghai, China. Diabetes patients were selected because they have been found in previous studies to be particularly vulnerable to ambient stimulus due to their inherent high inflammatory state.^14^, ^15^, ^16^, ^17^, ^18^

## Methods

### Subjects and study design

A total of 35 T2DM patients were recruited from an urban community (Tianping) of Shanghai, which has an area of 2.6 km^2^ and a population of 86,000. Only doctor-diagnosed T2DM patients who had a permanent residence in Tianping Community were recruited in this study.^19^ We excluded those who were current active or passive smokers (at home), had an alcohol drinking habit, or were experiencing apparent cardiopulmonary comorbidities.^20^, ^21^, ^22^

Six follow-up visits were conducted every 2 weeks in Tianping Community Health Service Center during April to June of 2013 to capture day-to-day variations in both environmental and health indicators. For each participant, we scheduled health examinations at the same time of the day and in the same day of the week to avoid possible temporal variations. Baseline information, including age, sex, income, duration of T2DM, medication use, oral supplements, fast and postprandial blood glucose, and anti-diabetic medication, was collected using self-administered questionnaires. Height and weight were measured at the first follow-up to calculate the body mass index (BMI). We also asked all participants to recall their occupational exposure to PM, and to record any changes in medications and whether they went out of the central urban areas of Shanghai during the study period.

### FeNO measurements

The FeNO measurements were obtained using a portable NIOX MINO machine (Aerocrine AB, Solna, Sweden) according to the standardized procedures by the American Thoracic Society.^3^ Briefly, subjects were required to sit quietly, rinse the mouth twice with purified drinking water, and then empty theirs lungs by complete expiration. After that, they inhaled nitric oxide-free air to the lung capacity through a disposable filter attached in the machine. Finally, they exhaled the air through the machine at an exhalation rate of 50 ± 5 mL/s. The standard mode of 10-second exhalation time was used in all tests. To maintain a stable flow during exhalation, the participants were guided by an exhalation flow-driven animation. Quality control was automatically performed, and the device would not display a reading if the subject exhaled at a flow rate below or above the required speed. All our participants were asked to avoid eating and drinking for at least 1 h before the test.

### Exposure measurements

We obtained hourly concentrations of PM_2.5_ and PM_10_ from a fixed-site air quality monitoring station, which was approximately 2.5 km away from the Tianping community. We calculated the real-time concentrations of PM_10-2.5_ by subtracting PM_2.5_ from PM_10_ concentrations measured in the same hours. Both PM_2.5_ and PM_10_ were measured using the tapered element oscillating microbalance method. According to the rules of Chinese government, the location of this monitor is mandated not to be in the direct vicinity of traffic or industrial sources; not to be influenced by local pollution sources; and to avoid buildings, housing, and large emitters such as coal-, waste-, or oil-burning boilers, furnaces, and incinerators. Likewise, there were no apparent emission sources, such as industry and trunk roads, within and around this urban community. Therefore, the monitoring data may well represent the background air pollution level in urban Shanghai, as well as the general exposure levels of the study participants.

Hourly temperature and relative humidity data were derived from a monitoring station of Shanghai Meteorology Bureau, which was approximately 2 km from this community.

### Statistical analysis

Environmental exposure data were linked with FeNO values for each participant by the time of each test. We used mixed-effects models combined with the distributed lag nonlinear model (DLNM) to estimate the short–term associations of temperature and PM with FeNO levels. The mixed-effects model allows each participant to serve as his or her own control and allows adjustment of variations in repeated measurements for the same subject. The DLNM has the advantage of accounting for the possibly nonlinear effects of an exposure and the colinearity of different lags.^23^, ^24^ FeNO levels were naturally log-transformed because they were right skewed, and they were then introduced into the model as a dependent variable.

Briefly, we first built the cross-basis functions in each DLNM for temperature and PM. We used a natural cubic spline function with three equally spaced internal knots for temperature and a linear function for PM in the exposure-response functions. We introduced a natural cubic spline function with two internal knots at equally spaced log-values of lags in the cross-basis functions. The referent for temperature was set as the point with minimum effects on FeNO, and the referent for PM was set as the lowest concentrations during the study period. Thereafter, we incorporated the cross-basis functions in the mixed-effect models, with adjustment for individual characteristics (i.e., age, sex, BMI, income, and duration of diabetes), relative humidity, temperature (for PM models), and day of the week. Finally, a random intercept for each subject was added to account for correlations among multiple repeated measurements. In order to explore the lag structures, we used a maximum lag up to 10 days for temperature and up to 7 days for PM. We also performed a sensitivity test for all 198 FeNO measurements collected during the study period from 33 participants by adjusting the calendar days for seasonality in basic models of temperature and PM, separately.

In order to evaluate the potential modifying effect of temperature in the association between PM and FeNO, we conducted a stratified analysis by dichotomizing the study period into warm and cool days and then examining the associations between PM_2.5_ or PM_10-2.5_ and FeNO using the above models.

All statistical tests were two-sided, and *P* values <0.05 were considered statistically significant. The models were constructed in R software (Version 3.2.0, R Foundation for Statistical Computing, Vienna, Austria) with the packages ‘*lme4*’ and ‘*dlnm*’. The results for temperatures were presented as the relative risks (RRs) comparing a given temperature to the referent temperature because a nonlinear relationship was hypothesized. The results for temperatures were presented as RRs of FeNO comparing a given value to the referent temperature. The results for PM were presented as percentage increase in FeNO per 10 μg/m^3^ increase in PM concentrations.

### Ethical approvals and informed consent

The Institutional Review Board of the School of Public Health, Fudan University approved this study, and all participants provided written informed consent at the enrollment.

## Results

### Data description

Overall, 33 of the 35 participants finished all the scheduled tests. Table 1 summarizes the demographic characteristics of our participants at baseline. The number of male subjects was nearly equal to the females. The average age was 66 years, and 26 participants took regular anti-diabetic medications, mainly metformin and sulfonylureas. The duration of T2DM ranged from 4 to 13 years, with an average of 9 years. No subjects reported a history of occupational PM exposure or allergy. During the study period, no subjects left the urban areas of Shanghai in 3 days before each scheduled test, and none reported an occurrence of respiratory infection or atopic symptoms during the week before each test.

**Table 1:** Basic characteristics of the study participants.

	Mean (SD)
Number	33
Male	17
Age, years	65.9 (8.4)
Height, cm	164.3 (6.9)
Weight, kg	69.8 (9.6)
BMI, kg/m^2^	25.9 (3.5)
Annual income	
<¥50,000	22 (66.6%)
¥50,000–¥100,000	10 (30.3%)
>¥100,000	1 (3.0%)
Duration of T2DM, years	9.8 (8.7)
Fasting blood glucose, mmol/L	7.2 (1.6)
Postprandial blood glucose, mmol/L	10.0 (2.2)
Glycosylated hemoglobin, %	7.5 (1.0)

BMI, body mass index; T2DM, type 2 diabetes mellitus; SD, standard deviation.

We completed a total of 198 FeNO tests (33 × 6), with a mean result of 22.3 ppb (Table 2). Table 2 also provides summary statistics on personal exposure variables. The 24-h mean concentrations of PM_2.5_ and PM_10-2.5_ were 47.1 μg/m^3^ and 23.6 μg/m^3^, respectively. The average temperature and relative humidity were 21.6 °C and 71.7%, respectively, reflecting the subtropical climate in Shanghai.

**Table 2:** Summary of FeNO, 24-h mean concentrations of particulate matters, and 24-h average weather conditions.

	Mean (SD)	Min	Median	Max	IQR
FeNO, ppb	22.3 (15.3)	4.0	17.0	90.0	17.5
Particulate matter, μg/m^3^					
PM_2.5_	47.1 (21.6)	12.2	44.1	106.7	33.8
PM_10-2.5_	23.6 (21.9)	2.1	17.4	104.7	20.2
Temperature, °C	21.6 (4.8)	8.7	21.4	32.8	6.7
Relative Humidity, %	71.7 (14.8)	40.0	73.0	93.0	18.0

FeNO, fractional exhaled nitric oxide; IQR, interquartile range; PM_2.5_, particulate matter with an aerodynamic diameter ≤2.5 μm; PM_10-2.5_, particulate matter with an aerodynamic diameter between 2.5–10 μm; SD, standard deviation.

Both PM_2.5_ and PM_10-2.5_ were negatively associated with ambient temperature (Pearson r = −0.07 for PM_2.5_, and −0.29 for PM_10-2.5_) and relative humidity (Pearson r = −0.31 for PM_2.5_, and −0.80 for PM_10-2.5_).

### Regression results

**Fig. 1:**
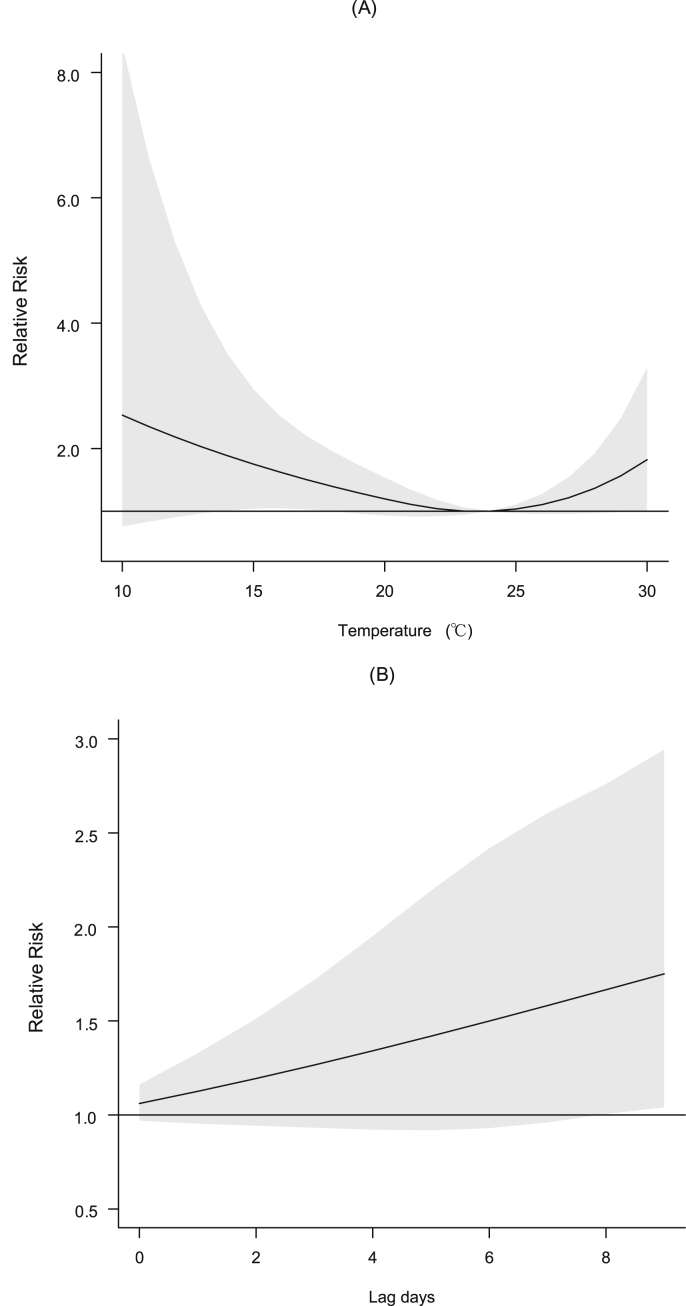
Dose-response relationships (A, lag 0–9 days) and cumulative lag patterns (B, 0–9 days) for the relative risk comparing a given temperature (15 °C) to the referent temperature (24 °C). The X-axis refers to temperature; the Y-axis refers to relative risk in FeNO relative to the referent temperature. The black lines are the mean relative risks, and the gray areas are the 95% confidence intervals of risk estimates.

As shown in Fig. 1, there were nonlinear associations between ambient temperature and FeNO (see Fig. 1), with a referent temperature of 24 °C. The curve had widened confidence intervals (CIs) at the lower end of the temperature distribution. Higher temperatures than 24 °C are associated with elevated levels of FeNO, but the increase was not statistically significant. For example, at lags of 0–9 days, the cumulative RR at 15 °C to the referent temperature was 1.75 (95% CI, 1.04–2.94). In addition, our sensitivity test of controlling calendar days for seasonality did not show substantial changes to the effect estimate of 15 °C (RR 1.76; 95% CI, 1.04–2.98) compared with our main results in the basic model.

**Fig. 2:**
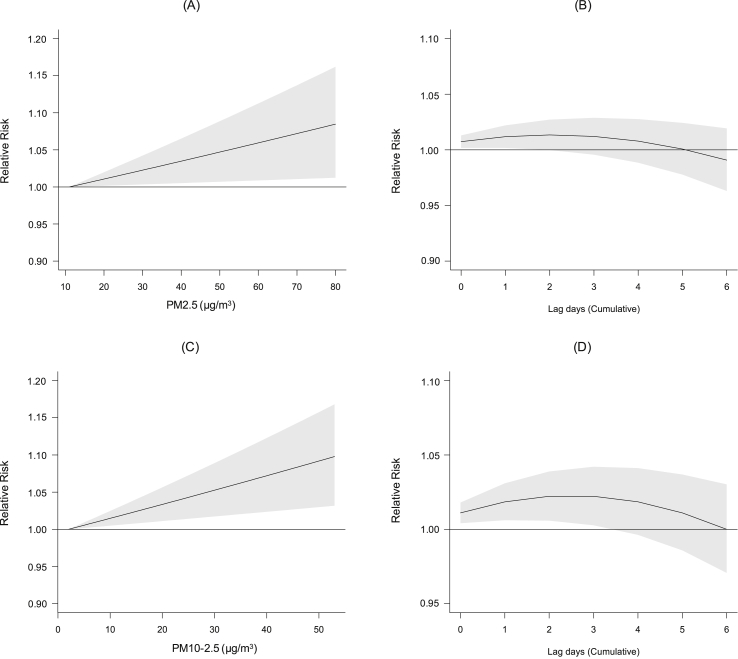
Dose-response relationships (A and C, lag 0–1 days) and lag patterns (B and D, 0–6 days) for the relative risk per 10 μg/m^3^ increase in PM_2.5_ and PM_10-2.5_. The X-axis refers to PM concentrations; the Y-axis refers to the relative risk per 10 μg/m^3^ increase in PM concentrations. The black lines are the mean relative risks, and the gray areas are the 95% confidence intervals of risk estimates. PM_2.5_, particulate matter with an aerodynamic diameter ≤2.5 μm; PM_10-2.5_, particulate matter with an aerodynamic diameter between 2.5–10 μm.

Both PM_2.5_ and PM_10-2.5_ were positively associated with FeNO, but the associations of PM_2.5_ were restricted to lag 0–1 days and then lost statistical significance with longer lags (2–6 days), while the associations of PM_10-2.5_ lasted until lag 0–3 days (Fig. 2). For example, the cumulative increments in FeNO were 1.18% (95% CI, 0.18–2.20) and 1.85% (95% CI, 0.62–3.09) per 10 μg/m^3^ increase in PM_2.5_ and PM_10-2.5_, respectively, over lag 0–1 days. Additional adjustment for seasonality in the sensitivity test showed no significant changes to the effects of PM_2.5_ (1.28%; 95% CI, 0.28–2.29) and PM_10-2.5_ (1.63%; 95% CI, 0.39–2.89) on FeNO compared with our main results in the basic model.

**Fig. 3:**
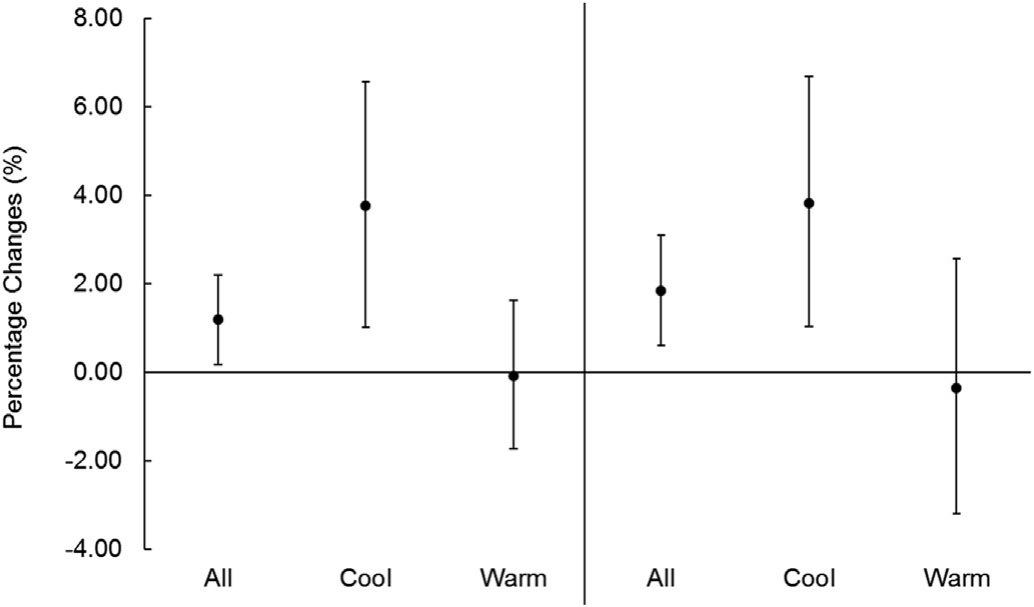
Percentage increase (means and 95% confidence intervals) in FeNO per 10 μg/m^3^ increase in 2-day average PM_2.5_ and PM_10-2.5_ concentrations under cool (≤20.7 °C) and warm (>20.7 °C) temperatures. PM_2.5_, particulate matter with an aerodynamic diameter ≤2.5 μm; PM_10-2.5_, particulate matter with an aerodynamic diameter between 2.5–10 μm.

Fig. 3 provides the effect estimates of PM (cumulative lags of 0–1 days) in cool days and warm days by dichotomizing the 2-day average temperature with a median cut-off of 20.7 °C. Both PM_2.5_ and PM_10-2.5_ had much stronger effects in cool days than in warm days, and the stratified effect size was nearly the same for fine and coarse PM. For example, FeNO increase by 3.76% (95% CI, 1.02–6.58) and 3.83% (95% CI, 1.04–6.69) per 10 μg/m^3^ increment in PM_2.5_ and PM_10-2.5_, respectively, in cool days, while the corresponding increases were −0.07% (95% CI, −1.73 to 1.62) and −0.35% (95% CI, −3.19 to 2.57) in warm days.

## Discussion

This study demonstrated significant associations of short-term exposure to ambient low temperature and PM with FeNO levels among a panel of T2DM patients in Shanghai, China. Further, temperature may act as an effect modifier in the association between PM and FeNO. To our knowledge, our study is one of very few studies that provide epidemiological evidence regarding the association between low temperature and airway inflammation. Our results also add to the limited evidence about the adverse effects of PM on airway inflammation in China.

Although a number of epidemiological studies have observed the short–term associations between low ambient temperature and respiratory mortality or morbidity, the underlying mechanisms remain unknown. Respiratory inflammation constitutes a very important pathway by which exogenous stimulus affects the airway.^16^, ^17^, ^22^ Our results demonstrated an inverse association between temperature and FeNO. However, few studies have explored the association between ambient temperature and respiratory inflammation, and the results have been inconsistent. For example, Bhowmik and colleagues observed a higher level of FeNO in the cold season among a cohort of chronic obstructive pulmonary disease (COPD) patients, but failed to directly evaluate the effects of temperature.^25^ Another study revealed that cold exposure was significantly associated with sputum neutrophils counts (another indicator of airway inflammation), but not with FeNO, in patients with asthma.^26^ Given the very limited evidence in this area, further studies are needed to confirm our findings.

The association between low temperature and FeNO appeared to be biologically plausible. First, exhaled nitric oxide is generated by inducible nitric oxide synthase (iNOS), which could be activated by both endogenous mediators, such as inflammatory cytokines, and exogenous factors, such as bacterial or viral infection.^27^ Epidemiological evidence has linked cold weather with higher prevalence of viral and bacterial infection in the respiratory tract, which can lead to inflammation and activation of iNOS, consequently resulting in higher NO production in the respiratory tract.^28^ Second, low ambient temperature can directly induce airway inflammation by increasing the counts of airway inflammation cells, which enhances the production of NO in the respiratory tract.^29^ Third, low ambient temperature may directly up-regulate the activity of iNOS by inducing DNA hypomethylation in the iNOS gene. A recent study among 777 elderly participants suggested a significant association between decreased temperature and hypomethylation of the iNOS gene.^30^ Fourth, decreases in temperature may activate airway transient receptor potential channels, such as TRPA1 and TRPM8, which could produce inflammatory cytokines and subsequently stimulate NO production in the respiratory tract.^31^ However, the biological mechanisms behind the associations of temperature and FeNO still need further investigation.

PM_2.5_ and PM_10-2.5_ levels in China are much higher than those commonly reported in North America and Western Europe, but evidence concerning the association of PM with FeNO has been limited.^32^ We observed significant short–term associations of PM_2.5_ and PM_10-2.5_ with FeNO levels, which were consistent with the broad literature of similar panel study designs. For example, a 10 μg/m^3^ increase in 48-h mean PM_2.5_ was associated with an increase of 2.12 ppb in FeNO in 37 diabetic patients in the United States.^33^ An interquartile range increase in traffic-related PM_10-2.5_ could lead to an elevation of 0.8 ppb.^34^ A panel study of 23 COPD patients conducted in Beijing, China also revealed significant increases in FeNO of 13.6% (95% CI, 4.8%–23.2%) per interquartile range increase (76.5 μg/m^3^) of PM_2.5._^19^

The significant associations between PM and FeNO was biologically plausible. First, iNOS DNA hypomethylation associated with short-term exposure to PM may increase the production of nitric oxide.^35^, ^36^ Second, inhalation of PM can trigger a number of redox-sensitive signaling cascades through oxidative stress-induced damage, consequently causing the exacerbation of airway inflammation and release of nitric oxide.^9^ Third, both *in vivo* and *in vitro* studies have demonstrated the oxidative potential of PM,^37^, ^38^ resulting in the activation of iNOS. Additionally, the different time lags we observed in the effects of PM and temperature suggests that the responses to PM and temperature may happen independently. Furthermore, we fit a two-pollutant model to evaluate the independent effects of coarse and fine PM with adjustment of each other but obtained insignificant estimates for both pollutants (data not shown). However, the results from bi-pollutant models should be interpreted with caution because of the relatively small sample size and the strong correlation between coarse and fine PM.

Our stratification analysis showed that temperature, in addition to influencing FeNO directly, may also interact with the association between PM and FeNO. However, the difference was statistically insignificant, probably due to the small sample size. The interactions between temperature and PM have also been reported in some previous studies, but the results have differed according to the study design, sample size, and outcomes. For example, Berhane et al found that the effects of PM_2.5_ and PM_10_ on FeNO were higher in warm seasons, probably because of higher concentrations in warm days.^7^ Studies in Korea and Hong Kong suggested higher respiratory mortality under low temperatures.^39^, ^40^ However, a study in Beijing, China reported higher PM_2.5_-related respiratory mortality under higher temperatures.^41^ The interaction between low temperature and PM might also be plausible. First, PM concentrations were always higher in cool seasons than in warm seasons. Second, previous studies indicated that cool air could reduce beat frequencies in nasal and trachea cilia, which may result in reduced clearance of inhaled PM.^42^ Third, both low temperature and PM can impair the respiratory tract through the same pathways, such as inflammation.

Our study had several limitations, and our results should be interpreted with caution. First, exposure measurement errors were inevitable in this study because we obtained data from a nearby fixed-site monitor as surrogates for personal exposure levels. However, we did not think that the errors had led to substantial bias in our findings because the distance (about 2.5 km) between the fixed-site monitor and the community was not large and no participants left the community during the study. Second, the sample size in our study was relatively small, which may add uncertainty to our results and limit the statistical power in our stratification analysis. Third, limited by data availability, we were unable to exclude the confounding effects of allergens. According to a previous report in Shanghai, airborne pollen level increased in similar months (from March to May) as those evaluated in our study.^43^ However, the failure to control for airborne pollen was not likely to exaggerate our results because temperature was increasing and PM concentrations were decreasing during the study period (from April to June). Besides, since no participants reported allergies during the study period, we believe that the daily fluctuation in allergens may not be a major concern in this study. Fourth, the use of multi-pollutant models in the present study was limited due to the correlations between particulate matters and gaseous pollutants and the dual measurement errors in relation to fixed-site monitoring. Fifth, the dual exposure measurement errors for temperature and particulate matters might make our results for the interactions more unstable, so these results should be cautiously interpreted.

In summary, this study suggested low ambient temperature, PM_2.5_, and PM_10-2.5_ may elevate the levels of respiratory inflammation among T2DM patients in Shanghai, China. Furthermore, the effects of PM were more evident when temperature was low. Our findings may help understand the epidemiological evidence linking ambient temperature, particulate air pollution, and respiratory health. Further investigations with larger sample sizes and personal exposure measurements are needed to confirm our findings.

## Conflicts of interest

None declared.
